# Magnesium Ingot Stacking Segmentation Algorithm for Industrial Robot Based on the Correction of Image Overexposure Area

**DOI:** 10.3390/s23156809

**Published:** 2023-07-30

**Authors:** Qiguang Li, Huazheng Zheng, Wensheng Wang, Chenggang Li

**Affiliations:** 1 School of Mechanical and Electrical Engineering, Beijing Information Science and Technology University, Beijing 100192, China; 2 Jiaxing Worldia Diamond Tools Co., Ltd., Jiaxing 314031, China

**Keywords:** magnesium ingot sorting, the high reflection of magnesium ingot, exposure correction, magnesium ingot segmentation

## Abstract

This paper proposes an adaptive threshold segmentation algorithm for the magnesium ingot stack based on image overexposure area correction (ATSIOAC), which solves the problem of mirror reflection on the surface of magnesium alloy ingots caused by external ambient light and auxiliary light sources. Firstly, considering the brightness and chromaticity information of the mapped image, we divide the exposure probability threshold into weak exposure and strong exposure regions. Secondly, the saturation difference between the magnesium ingot region and the background region is used to obtain a mask for the magnesium ingot region to eliminate interference from the image background. Then, the RGB average of adjacent pixels in the overexposed area is used as a reference to correct the colors of the strongly exposed and weakly exposed areas, respectively. Furthermore, in order to smoothly fuse the two corrected images, pixel weighted average (WA) is applied. Finally, the magnesium ingot sorting experimental device was constructed and the corrected top surface image of the ingot pile was segmented through ATSIOAC. The experimental results show that the overexposed area detection and correction algorithm proposed in this paper can effectively correct the color information in the overexposed area, and when segmenting ingot images, complete segmentation results of the top surface of the ingot pile can be obtained, effectively improving the accuracy of magnesium alloy ingot segmentation. The segmentation algorithm achieves a segmentation accuracy of 94.38%.

## 1. Introduction

With its excellent properties such as low density, high specific strength and specific stiffness, and good thermal conductivity, magnesium alloy is widely used in robotics [1,2], image processing [3,4], automotive, aerospace, and communication electronics, and also plays an increasingly important role in realizing the light weight of products [5,6,7]. During the magnesium alloy melting process, the key to achieving magnesium ingot sorting with the magnesium ingot automatic picking equipment is the segmentation of the magnesium ingot image. And the quality of the segmentation results will affect the grasping efficiency of the subsequent magnesium ingot automatic loading equipment. Therefore, the segmentation of magnesium ingots in images has important research value.

**Figure 1:**
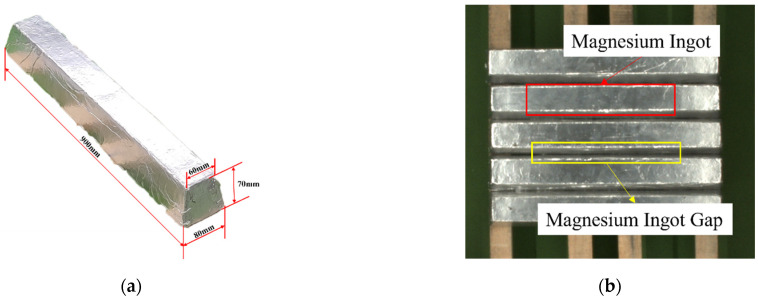
Schematic diagram of magnesium ingot and magnesium ingot gap: (**a**) magnesium ingot; (**b**) magnesium ingot gap.

The segmentation object in this paper is a magnesium ingot with a trapezoidal cross-section, and the specific dimensions are shown in Figure 1a. The surface of the target magnesium ingot is smooth and local overexposure will occur in the acquired image under a complex external light environment [8]. When the image is segmented, the gradient of partial gray value information of the magnesium ingot changes dramatically, and the segmentation result of the magnesium ingot obtained by traditional methods is incomplete. When magnesium ingots are tightly arranged, there are gaps between the top surfaces of the ingots due to their trapezoidal cross-section. The color of the gaps is similar to that of the top surfaces of the ingots. During the process of the magnesium ingot segmentation, these gaps can interfere with the segmentation results, leading to suboptimal image segmentation outcomes. The schematic diagram is shown in Figure 1b.

Currently, in the industrial field, machine vision [9] technology has been widely applied to image segmentation of metal workpieces to better improve industrial production efficiency [10,11,12]. The high reflectivity of metals is a common problem that affects the effectiveness of segmentation algorithms. Jiang et al. [13] established an experimental platform that is composed of a linear array camera, a linear light source, an encoder, and a rotation system to improve image quality in response to the phenomenon of uneven illumination of images caused by the high reflection characteristics of steel pipes. Furferi et al. [14] designed three different lighting environments for the high reflectivity of small metal parts to obtain images that are more conducive to segmentation processing. Feng et al. [15] proposed an adaptive local tone mapping algorithm based on gradient domain to solve the problem of local oversaturation of highly reflective metal surfaces, reducing color shifts caused by brightness compression, enhancing color perception of images, and compensating for the differences between display devices and natural scenes under visual conditions. Zhou et al. [16] used the BEMD algorithm to process images for high contrast textures caused by objective factors such as lighting in order to filter out noise and suppress surface textures. Qiu et al. [17] designed a dual weighted principal component analysis algorithm based on visual and spatial features to reduce the impact of high reflection on the surface of metal workpieces. Existing studies usually fail to locate overexposed regions accurately, and unrecognized overexposed regions may have new impacts on recognition or segmentation. Some studies focus on the identification of small parts, using filtering methods to repair overexposed areas, which are not suitable for large metal planes. Therefore, this paper proposes an accurate algorithm for detecting exposed regions, and based on this, color correction is performed based on neighborhood information to minimize the impact of overexposure on recognition or segmentation.

On the other hand, the disorderly placement of metal parts also requires high stability and accuracy of the algorithm. Hsu et al. [18] developed a machine vision system capable of automatic classification for different lighting environments. Under back-lighting and front-lighting environments, they used global and local threshold methods to achieve simultaneous segmentation of multiple metal parts. Li et al. [19] proposed a method for detecting and recognizing surface features of metal workpieces. Aiming at the problems of uneven illumination and reflection on the surface of metal workpieces, the Retinex algorithm was used for image enhancement, the Otus algorithm was used for the segmentation of target features on the surface of metal workpieces, and the vertical projection of a binary graph was used for the single separation of target features. Astain et al. [20] proposed a detection, recognition, and positioning system for reflective metal workpieces, using background subtraction and Viola–Jones classifiers to identify target workpieces. Chen et al. [21] used Rotated MS R-CNN and Mask R-CNN to achieve the segmentation of randomly overlapping screws. Zheng et al. [22] proposed a workpiece capture region recognition method that combines deep learning and support vector machines. The method used the Mask R-CNN neural network to complete the preliminary segmentation of the workpiece capture region and used an SVM classifier with multiple feature fusion to perform secondary fine segmentation of the Mask R-CNN recognition results, achieving the elimination of interference regions, and completing the recognition of the irregular placement of workpieces. The methods based on deep learning have high recognition accuracy, but it is difficult to collect data for training, and the results generated during the scene transformation are unstable. Some research is based on clustering or statistical threshold setting methods. They do not adapt to the complex situation of metal parts in space. Therefore, this paper proposes a method that combines the adaptive threshold segmentation algorithm and the morphological algorithm to segment the corrected image. This method has good robustness and can solve edge recognition errors between metal parts.

Currently, there is little research on large metal ingots such as magnesium ingots. Due to the large surface area of magnesium ingots, which are more susceptible to light effects, research on the segmentation and recognition of small metal workpieces is not applicable to magnesium ingots. In addition, the disorderly placement of metal workpieces may lead to difficulties in extracting complete edges. The industrial-grade 3D sensors are expensive, and when using depth cameras to capture images, the high reflective areas on the magnesium ingot surface cause depth information loss and create holes. Therefore, considering the comprehensive factors, this paper proposes an adaptive threshold magnesium ingot stack segmentation algorithm based on image overexposure region correction, using color cameras to capture images. This algorithm can quickly and robustly segment magnesium ingots in an image. The main contributions of this paper are as follows:

Firstly, a color correction algorithm based on overexposure detection is proposed. This algorithm accurately identifies the areas of magnesium ingot overexposure under various lighting conditions and corrects the color information of the target ingot. As a result, the grayscale gradient of the image becomes more uniform, leading to improved image quality. Secondly, based on this color correction algorithm, an adaptive threshold segmentation algorithm for stacking magnesium ingots is designed. This algorithm utilizes threshold segmentation and morphological processing techniques to segment the top surfaces of the corrected images of the ingots. It effectively addresses the issues of segmentation results sticking when magnesium ingots are arranged and distributed and the segmentation challenges caused by overexposure.

The other chapters of this article are described as follows: In the second section, the construction of the experimental light source environment is firstly introduced, then the overexposure area detection algorithm and color correction algorithm are introduced in detail for the experimental object. Finally, an adaptive threshold magnesium ingot stack segmentation algorithm is introduced, which is suitable for the image that the color of the overexposure area is corrected. In the third section, we quantitatively compare our algorithm with other image segmentation algorithms, and design multiple sets of controlled experiments to verify the reliability and accuracy of our algorithm. The fourth section summarizes the main contributions of this article and draws conclusions.

## 2. Materials and Methods

**Figure 2:**
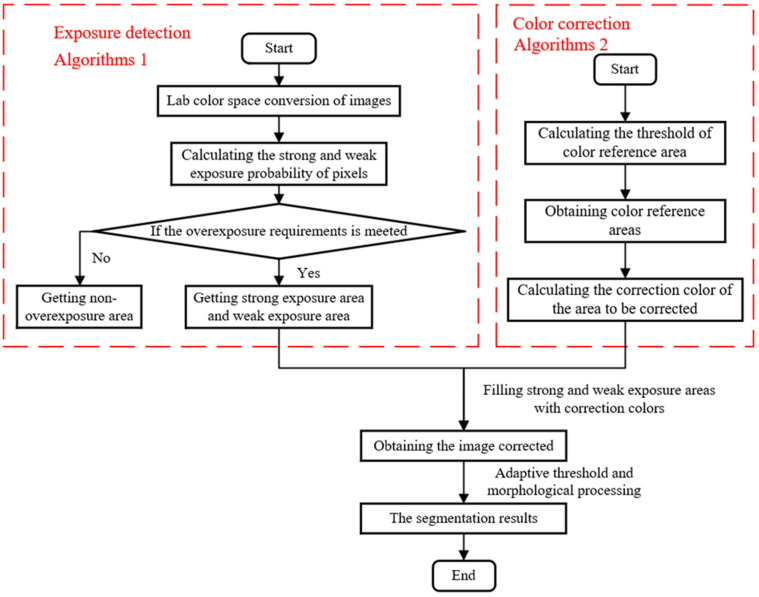
Overall algorithm flow.

This paper proposes an algorithm for detecting and correcting overexposed regions, and designs an adaptive threshold magnesium ingot stack segmentation algorithm based on image overexposed region correction, which can effectively solve segmentation failures caused by object overexposure. The algorithm flowchart is shown in Figure 2.

### 2.1. Light Source Environment Construction

In order to meet the high-quality requirements for the original image of the image recognition, in visual inspection systems, it is often necessary to design appropriate lighting methods with the help of professional visual light sources to illuminate the target object to be detected. Building a suitable light source environment can enhance the distinction between the target object and the background in the captured image, form an imaging effect conducive to image processing, obtain more information about the target object, and reduce the difficulty of image-processing algorithms [23,24].

**Figure 3:**
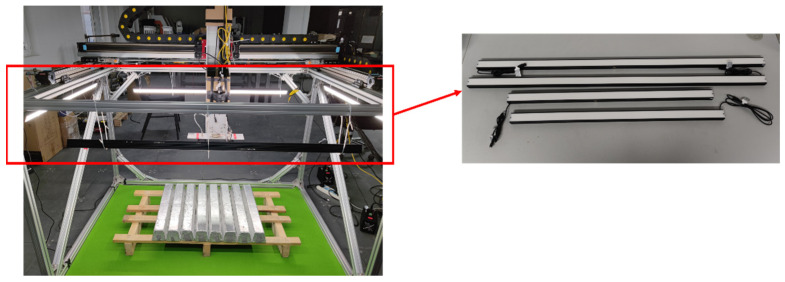
Schematic diagram of light source environment. The objects in the red box are the strip light sources used in the experiment. Their lengths are 800 mm and 1200 mm.

In natural environments, differences in time, weather, and the indoor environment can cause uneven brightness distribution in the target area in the captured image, directly affecting the segmentation results of the target object [25]. According to the structural characteristics of the target object and the equipment, this paper has built a light source environment composed of four strip light sources, as shown in Figure 3. The models are KM-BRD120030-W-24 and KM-BRD80030-W-24 from KOMA Vision.

**Figure 4:**
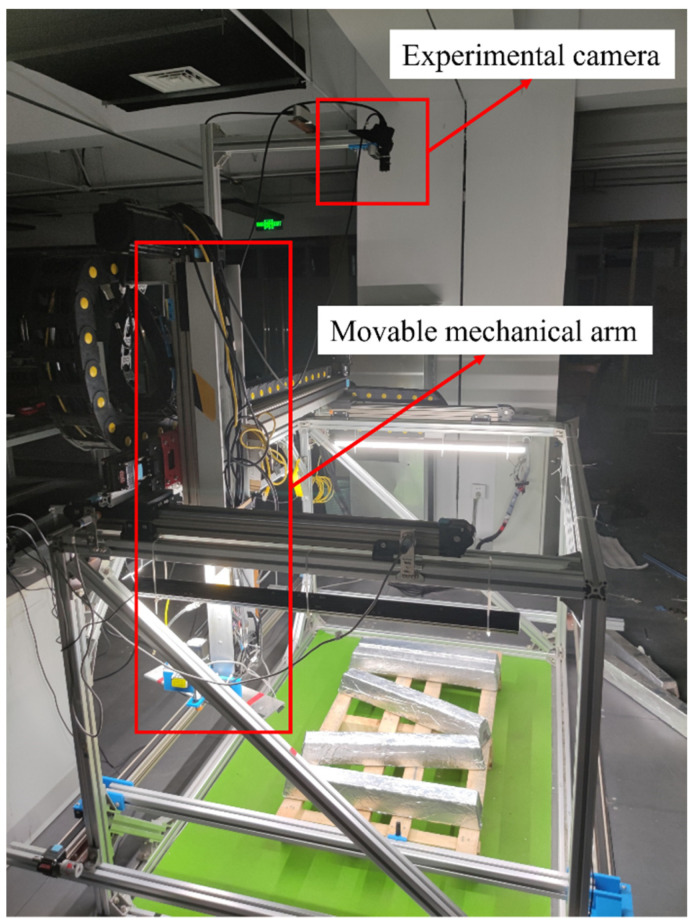
Schematic diagram of the installation position of the experimental camera and movable mechanical arm.

According to the arrangement of magnesium ingots and the frame structure of the equipment, two sizes and lengths of strip light sources, 800 mm and 1200 mm, are selected and placed symmetrically. They are surrounded by a high angle above the target magnesium ingot for illumination. In order to ensure a clean background for the captured image and to better highlight the target object, the target magnesium ingot is placed on a solid color background. The constructed light source environment has a simple structure and is convenient for debugging, which can more effectively save space without affecting the movement of the mechanical arm above the magnesium ingot. The surrounding lighting scheme can effectively prevent the impact of shadows generated by the movement of the robot arm on the captured image, and avoid uneven brightness caused by shadows generated on the captured image. Figure 4 is a schematic diagram of the installation positions of the experimental camera and the movable mechanical arm.

**Figure 5:**
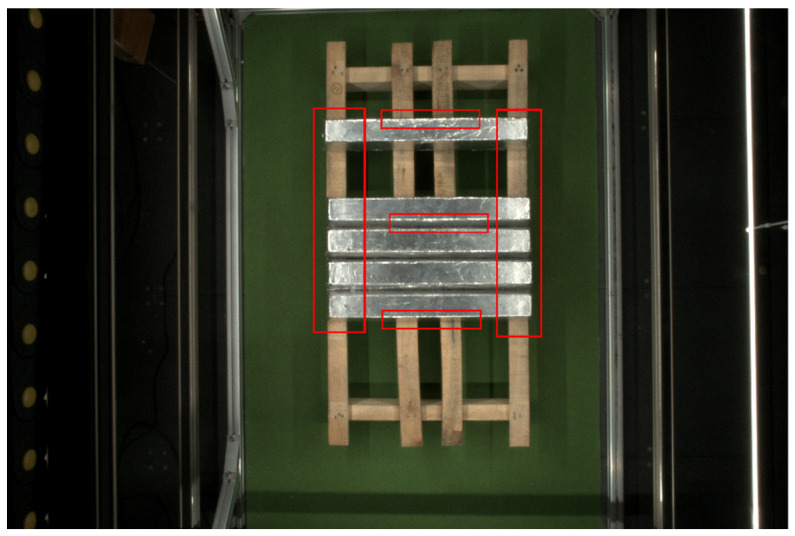
The acquisition result of the magnesium ingot image. The area within the box is the approximate overexposure area.

Because the magnesium ingot is located at the middle of the light source environment and the surface of the metal magnesium ingot is smooth, the parts of the magnesium ingot that are close to the light source may experience varying degrees of overexposure. However, the overexposure positions in the image are relatively concentrated, located at both ends of the magnesium ingot and at a few positions on the edge of the magnesium ingot, as shown in Figure 5.

### 2.2. Overexposure Area Detection Based on Lab Color Space

**Figure 6:**
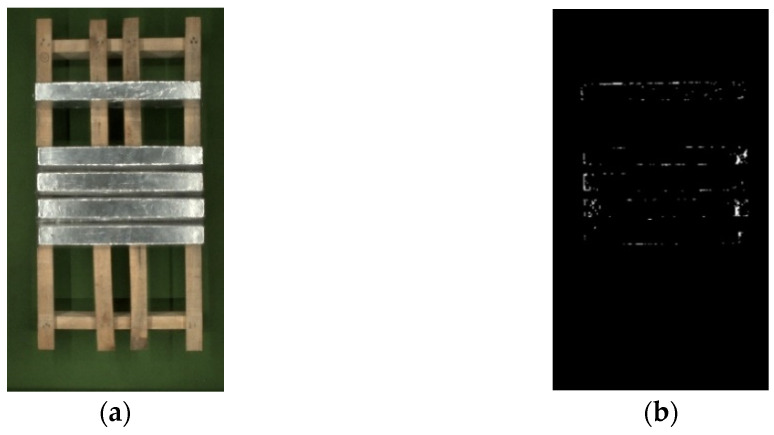
Results of the overexposure region segmentation based on the grayscale threshold: (**a**) magnesium ingot image; (**b**) gray threshold segmentation results.

We generally believe that the overexposed area in an image is white [26], and the grayscale value of this part in a grayscale image is 255. Using the grayscale threshold segmentation method, the part of the image with a grayscale value greater than 254 is defined as an overexposed area. Figure 6b shows the grayscale threshold segmentation result. As shown in Figure 6b, the segmented result is discrete and the region is not complete enough to handle the transition from the overexposed region to the adjacent region well.

In this paper, we use Lab color space to map the brightness and chrominance information in an image to determine whether there is a possibility of overexposure of pixels in the image, obtaining strong and weak exposure areas. Compared to traditional segmentation methods using single color information, this method can better handle the transition from overexposed regions to adjacent regions.

#### 2.2.1. Lab Color Space Conversion

Lab color space is a uniform color space model for visual perception, independent of device differences. It is a digital method for describing human visual perception based on physiological characteristics [27], where L represents the pixel brightness of the image, with a value range of [0, 100], and a and b represent the position of the pixel color in the color space, with a value range of [−127, 127]. This space is a three-dimensional rectangular coordinate system, and the color space model can be referenced in [27]. The three components are perpendicular to each other and correspond to x, y, and z in the coordinate axis based on the non-linear compressed XYZ color space coordinates.

Before converting an image from RGB color space to Lab color space, it is necessary to normalize the r, g, and b values of the image. The conversion relationship is shown in Equations (1) and (2):

(1) $$\{\begin{array}{c} R=gammma\left(\frac{r}{255}\right)\\ G=gammma\left(\frac{g}{255}\right)\\ B=gammma\left(\frac{b}{255}\right) \end{array}$$

(2) $$gamma(x)=\{\begin{array}{ll} {\left(\frac{x+0.055}{1.055}\right)}^{2.4} & ,x>0.04045\\ \frac{x}{12.92} & ,\mathrm{otherwise} \end{array}$$

In addition, the conversion between RGB color space and Lab color space is an indirect process, which requires mapping the RGB color space to the XYZ color space. The conversion relationship is shown in Equation (3).

(3) $${\left[\begin{array}{ccc} X & Y & Z \end{array}\right]}^{T}=A*{\left[\begin{array}{ccc} R & G & B \end{array}\right]}^{T},\;A=\left[\begin{array}{ccc} 0.4124 & 0.3575 & 0.1804\\ 0.2126 & 0.7151 & 0.0721\\ 0.0193 & 0.1191 & 0.9502 \end{array}\right]$$

Finally, the conversion of the XYZ color space to the Lab color space, and the conversion relationship is shown in Equation (4):

(4) $$\{\begin{array}{l} L=116f\left(Y/Y_{n}\right)-16\\ a=500\left[f\left(X/X_{n}\right)-f\left(Y/Y_{n}\right)\right]\\ b=200\left[f\left(Y/Y_{n}\right)-f\left(Z/Z_{n}\right)\right] \end{array}$$

(5) $$f\left(t\right)=\{\begin{array}{ll} t^{\frac{1}{3}} & ,t>{\left(\frac{6}{19}\right)}^{3}\\ \frac{1}{3}{\left(\frac{29}{6}\right)}^{2}t+\frac{4}{29} & ,x\geq 0 \end{array}$$

In Equation (4), $X_{n}$, $Y_{n}$, and $Z_{n}$ are related to the row vector elements of the transformation matrix A. The default values are 0.9504, 1.0, and 1.089.

#### 2.2.2. Overexposure Region Segmentation

The strong exposure area in the image has strong brightness, and just performing color correction on the strong exposure area will lead to uneven color transition of the magnesium ingot. Therefore, this paper divides the overexposed area in the image into a strong exposure area and weak exposure area, wherein the strong exposure area is the area that still has the possibility of overexposure in the weak exposure area.

We map the brightness and chroma information of the image I in the Lab color space and perform normalization processing. We use the brightness channels L and color channels C in the Lab color space to define the weak exposure region S in the image, which makes $C={\left[\begin{array}{cc} a & b \end{array}\right]}^{T}$. The weak exposure probability value $M_{i}$ of the pixel i is defined as:

(6) $$M_{i}=sigmoid\left(\delta \cdot \left(\gamma_{i}\cdot \left(L_{i}-L_{T}\right)+\left(C_{T}-{\left\|C_{i}\right\|}_{2}\right)\right)\right),i\in I,$$

where $L_{T}$ is the brightness boundary value of the overexposed area, $C_{T}$ is the chromaticity boundary value of the overexposed area, and δ controls the growth rate of $M_{i}$ when $L_{i}$ is larger or $\|C_{i}\|{}_{2}$. is smaller. $\gamma_{i}$ is a non-linear weight, as shown in Equation (7). It indicates that the greater the brightness, the higher the weight of the pixel in the overexposed area.

(7) $$\gamma_{i}=100\times {\left(\frac{L_{i}}{100}\right)}^{k},i\in I$$

The strong exposure area H is the area where there is still a possibility of overexposure in S. The strong exposure probability value $P_{i}$ of the pixel i is defined as:

(8) $$P_{i}=\frac{1}{g}\cdot \frac{1}{1-M_{i}},i\in I,$$

where g is the normalization factor to make $max_{i}P_{i}=1$.

**Figure 7:**
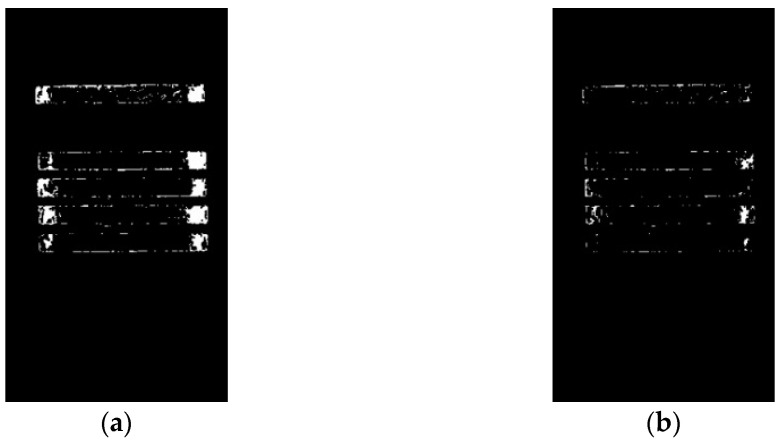
Overexposure region segmentation: (**a**) the segmentation area of the weak exposure region; (**b**) the segmentation area of the strong exposure region.

**Figure 8:**
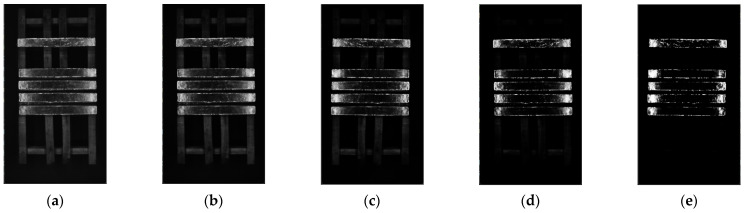
Result of image overexposure probability under different δ: (**a**) δ=1/50; (**b**) δ=1/40; (**c**) δ=1/30; (**d**) δ=1/20; (**e**) δ=1/10.

The critical value of weak exposure probability $d_{m}$ is 0.5. When $M_{i}\geq d_{m}$, it is indicated that there is a possibility of overexposure of the pixel, that is, overexposure occurs. After binarizing all overexposed pixels in the image, its result is shown in Figure 7a. The critical value of strong exposure probability $d_{p}$ is also 0.5. When $P_{i}\geq d_{p}$, it is considered that the region where the pixel is located is the area where there is still a possibility of overexposure in S, that is, the strong exposure region. The result is shown in Figure 7b:

In order to obtain the optimal parameters of δ, this paper selects five sets of values for comparison. When $\gamma_{i}=1$, it takes separately δ=1/50, δ=1/40, δ=1/30, δ=1/20, and δ=1/10. The results are shown in Figure 8. They show that as δ decreases, $M_{i}$ is less affected by the brightness and chromaticity of the exposed area, and the proportion of wooden frames in the background becomes larger, which will affect the selection of correction colors; but with the δ’s increase, $M_{i}$ gradually approaches the maximum value of 1, at which point $P_{i}$ is infinite, which will cause segmentation failure of the H region. Therefore, it is comprehensively considered to select δ=1/30.

**Figure 9:**
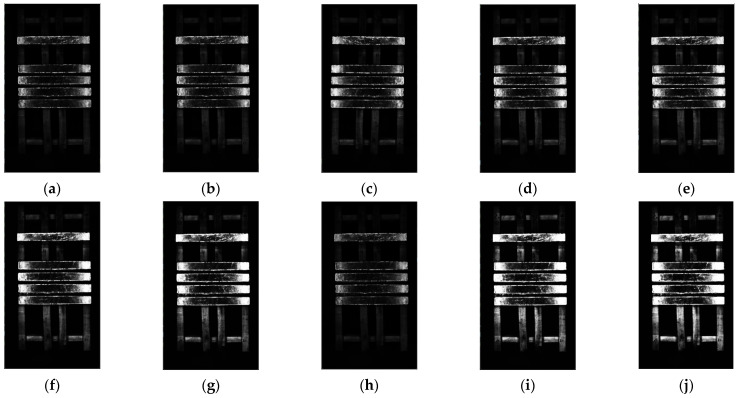
Results of image overexposure probability under different k: (**a**) k=0.1; (**b**) k=0.2; (**c**) k=0.3; (**d**) k=0.4; (**e**) k=0.5; (**f**) k=0.7; (**g**) k=0.9; (**h**) k=1; (**i**) k=1.2; (**j**) k=1.4.

$\gamma_{i}$ is a gamma function that serves as a non-linear weight to adjust the brightness information in the image to the impact of $M_{i}$. The index k is used as a variable in the weight, and its value is selected based on the optimal value obtained through multiple experiments for the object in this paper. We take k=0.1, k=0.2, k=0.3, k=0.4, k=0.5, k=0.7, k=0.9, k=1, k=1.2, and k=1.4 to compare the results. The comparison results are shown in Figure 9. When k increases, the impact of brightness information on $M_{i}$ becomes more severe, and the gradual disappearance of the brightness changes in the overexposed area. When calculating the H region, the infinite approximation of $M_{i}$ to 1 will result in the fact that the H region segmentation will fail. At this moment, $P_{i}$ is infinitely close to 0. The value of k is too small, resulting in incomplete segmentation of the S region. Therefore, it is comprehensively considered to select k=0.2.

The Pseudocode of the overexposure detection algorithm is shown in Algorithm 1.
**Algorithm 1:** Overexposure detection algorithm.**Input:** image I **Output:** strong exposure area S, weak exposure area H 1:  convert I to Lab color space 2:  **for** i∈I **do** 3:    calculate weak exposure probability value $M_{i}$, strong exposure probability value $P_{i}$ 4:    **if** 5:      $M_{i}\geq d_{m},\;P_{i}\geq d_{p},\;i\in I$ 6:      pixels i represent overexposure 7:    **else** 8:      pass 9:    **end if** 10:   **end for** 11:   $d_{m}\leftarrow \mathrm{Critical}\;\mathrm{value}\;\mathrm{of}\;\mathrm{weak}\;\mathrm{exposure}\;\mathrm{probability}$ 12:   $d_{p}\leftarrow \mathrm{Critical}\;\mathrm{value}\;\mathrm{of}\;\mathrm{strong}\;\mathrm{exposure}\;\mathrm{probability}$ 13:   **return** S, H

Compared to the segmentation by single threshold, the proposed method can segment more complete regions, including most of the regions in the image where brightness and chroma differ. In addition, the proposed method has a better ability to handle transition regions and obtain more complete segmentation results.

### 2.3. Overexposure Area Color Correction

Since the overexposed regions in the image are mainly concentrated at both ends and edge positions of the magnesium ingot, the region in the magnesium ingot whose color information is normal is used as a reference to determine the correction color, filling this color into the segmented overexposed region. Finally, the correction image of strong exposure and the correction image of weak exposure are fused according to the pixel WA [28].

#### 2.3.1. Acquisition of Magnesium Ingot Region Mask

Due to the shape and surface physical characteristics of magnesium ingots, which are affected by the surrounding lighting and lighting methods, the overexposed areas in the image are mainly concentrated at both ends of the magnesium ingot. Therefore, to correct the color of the overexposed areas, it is necessary to obtain the appropriate color in the normal color area of the magnesium ingot. First, it is important to eliminate the background effect in order to better obtain the appropriate color of the magnesium ingot.

**Figure 10:**
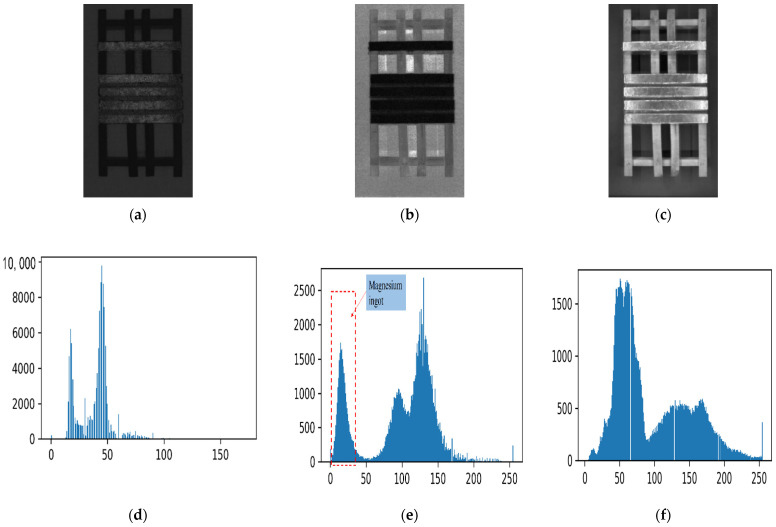
Two-dimensional images and histograms of three channel of HSV color space: (**a**) H channel image; (**b**) S channel image; (**c**) V channel image; (**d**) H channel binary image histogram; (**e**) S channel binary image histogram; (**f**) V channel binary image histogram.

The main objects in the collected images have significant color differences, and the HSV color space is used to segment the image background. After color space conversion of the image, the three channels of the HSV color space are separated and represented as binary images. Histograms of the three channels are drawn, as shown in Figure 10. In Figure 10a–c, the horizontal bar in the middle is a magnesium ingot, while the remaining objects are a wooden pallet and solid colored ground backgrounds.

**Figure 11:**
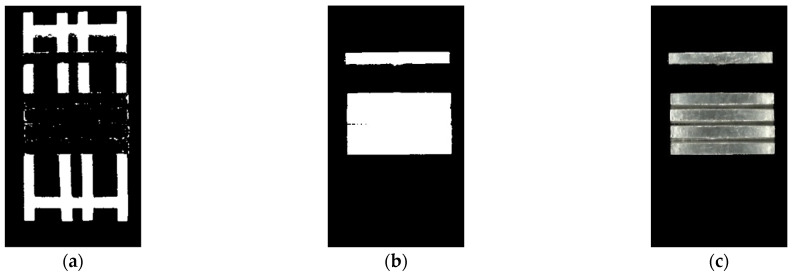
The results of mask acquisition and mask action results: (**a**) the threshold segmentation results of H channel binary image; (**b**) the threshold segmentation results of S channel binary image; (**c**) mask result from S channel.

The threshold segmentation of images utilizes the “bimodality” characteristic of image histograms. The binary image histograms in Figure 10d,e have obvious “bimodality”, while the binary image histograms in Figure 10f do not have this characteristic. So, it does not meet the conditions for the threshold segmentation of images. In Figure 10a, the magnesium ingot region is not significantly distinguished from other background objects in terms of hue values. If threshold segmentation is performed using the lowest point value of the bimodal depression in the histogram of the H channel binary image in Figure 10d, it will result in incomplete segmentation of the magnesium ingot region and the image background. Figure 11a shows the threshold segmentation result of an H channel binary image. The magnesium ingot region is confused with a solid color background, which is detrimental to the subsequent determination of the correction color. Using pixel saturation information to perform threshold segmentation on the S channel binary image, the resulting segmentation result is shown in Figure 11b, which better preserves the magnesium ingot region in the original image.

The threshold segmentation result obtained from the S channel binary image, namely the magnesium ingot region mask, mask, is applied as a mask to the original image to remove the background object and retain only the magnesium ingot. The result is shown in Figure 11c.

#### 2.3.2. Determining the Correction Color and Filling of Overexposed Areas

The color correction of an image is a process of inferring and filling to the color and texture information of the region to be corrected based on known region information. The color of the overexposed area of the magnesium ingot region differs from the normal region of the middle portion, and there is a significant color transition from both ends to the middle. A secondary filling method is used for color correction of this portion. Different correction colors are used to fill strong and weak exposure areas, respectively. Compared to single large area filling, processing for areas with different overexposure levels can achieve a uniform color transition effect.

The reference area for color correction is obtained from the exposure probability values of the underexposed pixels in the magnesium ingot. Taking color correction in the weakly exposed areas as an example, the algorithm is detailed as follows:

*A.*  Calculating the threshold of color reference area.

Using the idea of mask superposition, the original image is overlaid with the non-magnesium ingot region mask, $\bar{mask}$, to obtain the non-magnesium ingot region N. According to Equation (6), the weak exposure probability value, $M_{j}$(j∈N) of pixel j in N is calculated. The result of Equation (9) is the threshold $T_{1}$, which is as the left critical threshold for dividing the color reference region. Right critical threshold $T_{2}$ is $d_{m}$ from Section 2.2.2, that is $T_{2}=0.5$.

(9) $$T_{i}=\max\left(M_{j}\right),j\in N$$

*B.*  Obtaining color reference areas.

**Figure 12:**
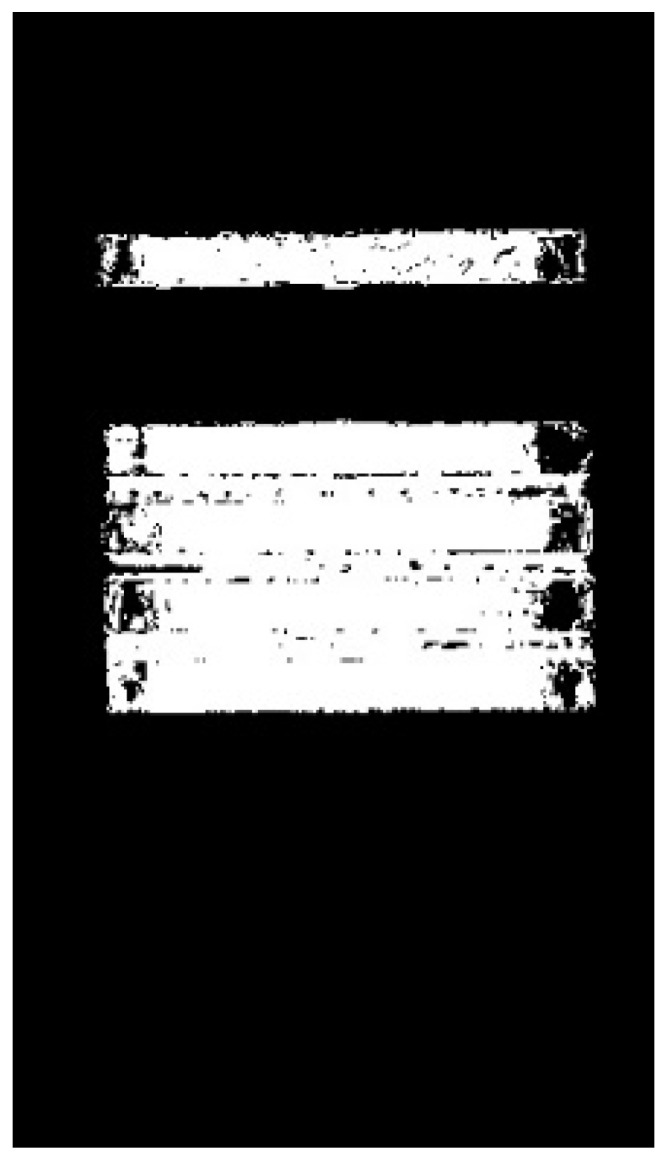
The portion of the magnesium ingot area that serves as a color reference. The white area is the area providing color reference. The white area is C, and based on this area, a color reference is provided for R.

We superposition the original image with mask to obtain the magnesium ingot region Q. If the pixel l within Q satisfies: $T_{1}\leq M_{l}\leq T_{2},l\in Q$, it is indicated that the region where the pixel is located can provide a color reference for the overexposed region, and it is recorded as the reference region C. If the pixel l satisfies: $M_{l}\geq T_{2},l\in Q$, it is considered that the region where the pixel is located is overexposed, and it is recorded as the region to be corrected R. The binarized image obtained using two critical thresholds is shown in Figure 12.

*C.*  Calculating the correction color of the area to be corrected.

The filling color of the region to be corrected R is calculated using the RGB value of the pixel in the reference region C, and the calculation equation is shown in Equation (10).

(10) $$\left[\begin{array}{c} \bar{R}\\ \bar{G}\\ \bar{B} \end{array}\right]=\frac{1}{n}\left[\begin{array}{c} \sum_{l=0}^{n}R_{l}\\ \sum_{l=0}^{n}G_{l}\\ \sum_{l=0}^{n}B_{l} \end{array}\right],l\in Q,$$

where $\bar{R}$, $\bar{G}$, and $\bar{B}$ is the r, g, and b values of the filling color of the pixels in R, n represents the number of pixels in C, and $R_{l}$, $G_{l}$, and $B_{l}$ is the r, g, and b values of pixels within C.

*D.*  Filling color.

**Figure 13:**
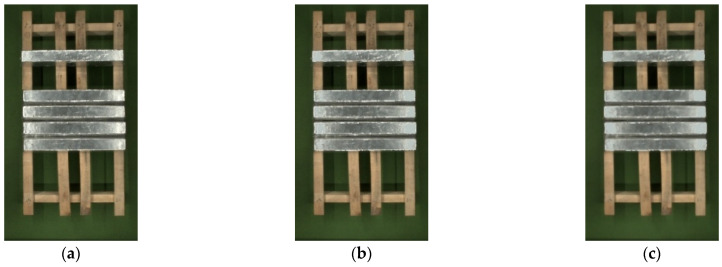
The results of color correction for weak exposure areas: (**a**) original image; (**b**) the result of color correction; (**c**) the result of median filter processing.

R is filled with the color that was obtained from Equation (10), and the result is shown in Figure 13b.

The Pseudocode of the color correction algorithm is shown in Algorithm 2.
**Algorithm 2:** Color correction algorithm (taking color correction for weak exposure as an example).**Input:** image I, S, H **Output:**
$\bar{R}$, $\bar{G}$, $\bar{B}$ 1:  convert I to HSV color space 2:  s_image ← the S channel image of image I 3:  mask← the result of threshold segmentation for s_image, represents the magnesium ingot area, and $\bar{mask}$ represents non-magnesium ingot region. 4:  N← the result of applying $\bar{mask}$ to image I 5:  Q← the result of applying mask to image I 6:  **for** j∈I **do** 7:    calculate the weak exposure probability value, $M_{j}\left(j\in N\right)\;\mathrm{of}\;\mathrm{pixel}\;j\;\mathrm{in}\;N$ 8:    $T_{1}$ is the Maximum value of $M_{j}$ 9:    calculate the weak exposure probability value, $M_{l}\left(l\in Q\right)\;\mathrm{of}\;\mathrm{pixel}\;l\;\mathrm{in}\;Q$ 10:     $T_{2}$ is $d_{m}$, critical value of weak exposure probability 11:     **if** 12:      $T_{1}\leq M_{l}\leq T_{2},l\in Q$ 13:      pixels l can provide a color reference for the overexposed region 14:      C is the reference region 15:     **else** 16:      pass 17:     **end if** 18:   $\bar{R}$, $\bar{G}$, $\bar{B}\leftarrow$ the average value of r, g, b of pixels in C 19:   **end for** 20:   **return**
$\bar{R}$, $\bar{G}$, $\bar{B}$

**Figure 14:**
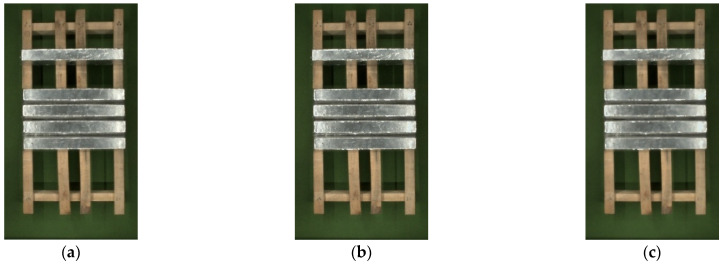
The results of color correction for strong exposure areas: (**a**) original image; (**b**) the result of color correction; (**c**) the result of median filter processing.

As can be seen from the Figure 13b, the color of the overexposed area has been improved. But after color filling, the filling edge appears jagged, and the color transition is uneven. A median filter is selected to filtering to blur its outline and interior and making the overall image color uniform, which achieves a smooth transition between high and low frequencies and protects edge information effectively. The result of median filter processing is shown in Figure 13c. The color correction algorithm for strong exposure areas is the same, and the results are shown in Figure 14.

#### 2.3.3. Image Fusion

**Figure 15:**
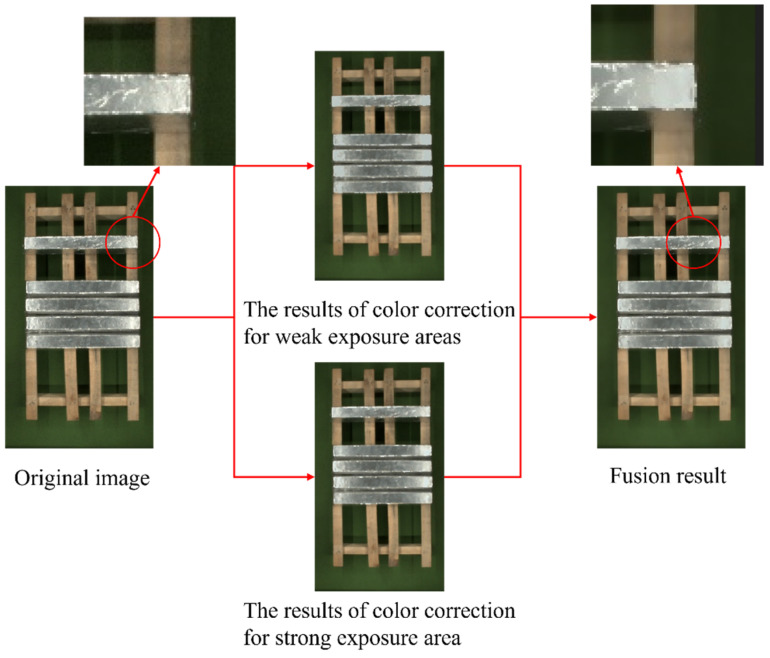
Image fusion results.

The image that is corrected by single exposure intensity has a certain degree of uneven color transition. From Section 2.2.2, we obtained the results of color correction for strong and weak exposure areas. According the rule of WA, the results are fused, as shown in Equation (11).

(11) $$dst=\alpha *src_{M}+\beta *src_{P}+\gamma ,$$

where dst represents an effect image after image fusion; α and β is image fusion weight, affecting the proportion of the image to be fused in the fusion result; $src_{M}$ and $src_{P}$ represents the result image after color correction for weak and strong exposure; and γ represents a scalar value attached to the sum of weights, and when γ becomes larger, the overall brightness value of the image becomes larger. For the object of this paper, α=0.5, β=0.4, and γ = 0, and the image fusion result is shown in Figure 15.

**Figure 16:**
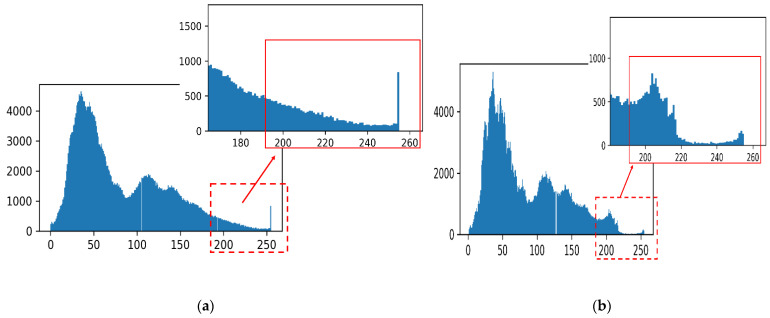
Histogram comparison before and after correction: (**a**) histogram of the original image; (**b**) histogram of color correction image.

Compared to the original image, the fusion result shows that the overexposure phenomenon on the magnesium ingot region is reduced and the color transition is uniform. From Figure 16, the number of pixels in the grayscale range 230–255 in Figure 16b is significantly less than that in Figure 16a, especially when the grayscale value is 255, which indicates that the overexposure area detection and correction algorithm proposed in this paper can effectively solve the overexposure phenomenon in the magnesium ingot region of the image.

### 2.4. Segmentation Algorithm Based on the Correction of the Image Overexposure Area

Compared to the original image, the brightness is more uniform and color information is more complete for the corrected image, which is conducive to the segmentation of magnesium ingot regions. In this paper, an adaptive threshold segmentation algorithm [29] is used to segment the magnesium ingot regions in the image. This method can adapt to complex and variable lighting environments compared to traditional threshold segmentation methods.

**Figure 17:**
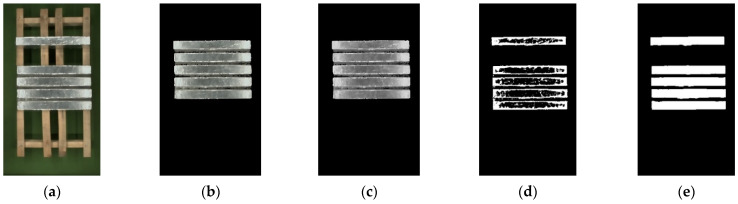
The results of image segmentation: (**a**) the result of color correction; (**b**) the result of mask superposition; (**c**) grayscale image; (**d**) the result of adaptive threshold segmentation; (**e**) the result of morphological processing.

The result of color correction in Section 2.3.3 is superimposed with mask obtained from Section 2.3.1. The results are shown in Figure 17b. It only retains the magnesium ingot region that narrows the segmentation range and reduces the computational complexity of the algorithm. Figure 17c is the result of grayscale conversion. The adaptive threshold segmentation function is run with select appropriate parameters and the results are shown in Figure 17d. The obtained result is processed by image morphology, and the result of the final segmentation is shown in Figure 17e.

## 3. Results and Discussion

**Figure 18:**
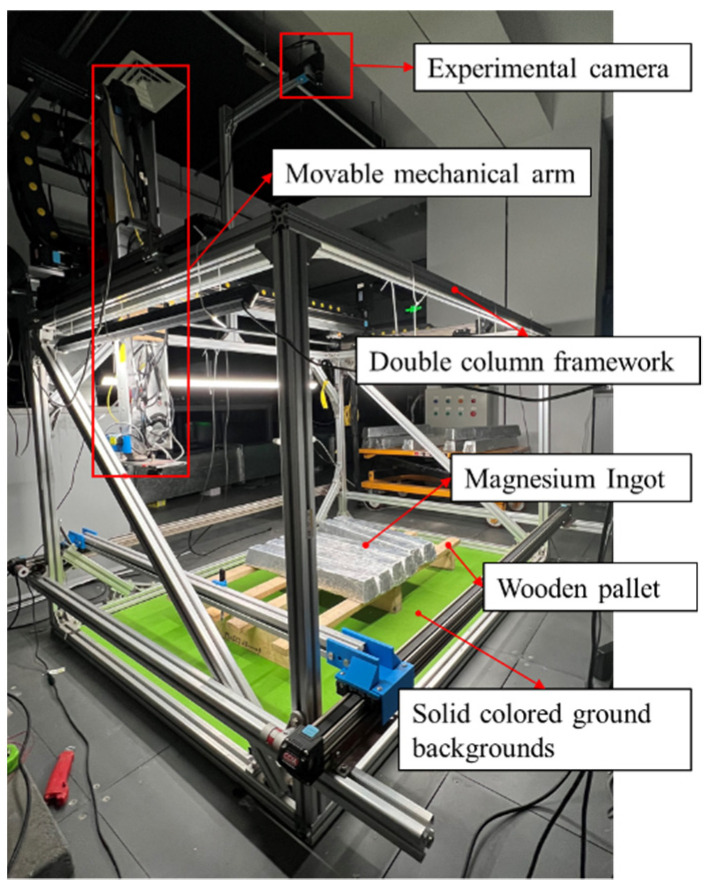
Magnesium ingot sorting device.

**Figure 19:**
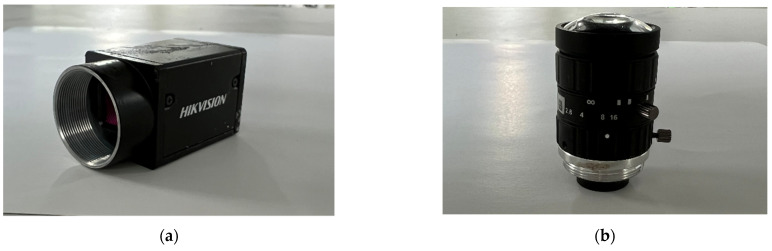
Experimental camera: (**a**) camera body; (**b**) camera lens.

To verify the image segmentation effect of the algorithm in this paper, experiments were conducted on an automatic magnesium ingot sorting device, as shown in Figure 18. Since there is overlap in the grayscale value range between the wooden frame area and the magnesium ingot area, the recognition of magnesium ingots cannot be achieved solely through the grayscale information of the images. Therefore, a color camera is chosen for image collection. The camera used in the experiment is Hikvision’s MV-CA060-10GC color camera, as shown in Figure 19a. The lens model used is Hikvision’s MVL-HF0628M-6MP wide-angle lens, as shown in Figure 19b. Its focal length is 6 mm and the image resolution is 3072 × 2048. The experiments shown in this paper mainly validate the algorithm from four aspects: (1) The impact of illumination on the algorithm when the magnesium ingot is at different positions; (2) the influence of light intensity on the algorithm; (3) the effectiveness of the algorithm under actual operating conditions; (4) the comparison between ours and other segmentation algorithms.

(1) Verifying the impact of illumination on the algorithm when the magnesium ingot is at different positions.

**Figure 20:**
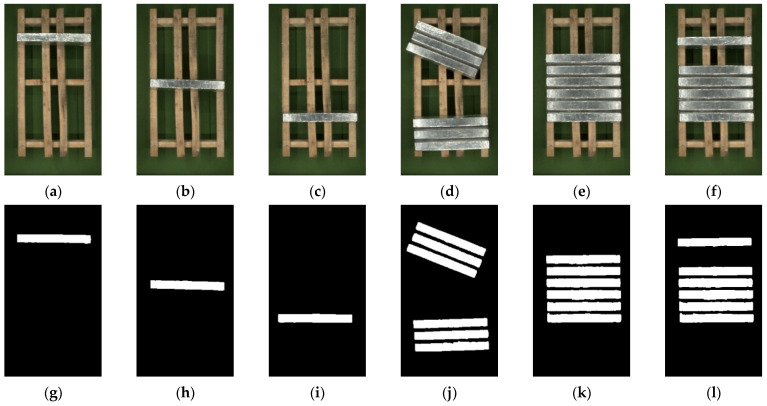
The results of image segmentation at different locations. (**a**–**f**) are the input images, and (**g**–**l**) are the results of image segmentation.

Due to the method of lighting on all sides, the distance between magnesium ingots at different locations and the light source is different, and magnesium ingots close to the light source are significantly affected by light, making overexposure more likely to occur. By controlling the number, placement position, and placement posture of magnesium ingots, the experiment was designed. And the segmentation results of magnesium ingots are shown in Figure 20.

In Figure 20, several images are selected with distinct positional characteristics and processed using the algorithm in this paper. The second row images are the segmentation results of images. In Figure 20a–f, when the magnesium ingot is located at different positions, the degree of overexposure on the surface will change. For the same magnesium ingot, both ends of the magnesium ingot are closer to the light source, so the positions of both ends of the magnesium ingot are significantly overexposed. When multiple magnesium ingots are arranged, the magnesium ingots located at the edge of the wooden tray are more prone to overexposure. From the results of image segmentation, whether it is changing the number of magnesium ingots or changing the placement position of magnesium ingots, the algorithm in this paper can achieve good segmentation results.

(2) Verifying the influence of light intensity on the algorithm.

**Figure 21:**
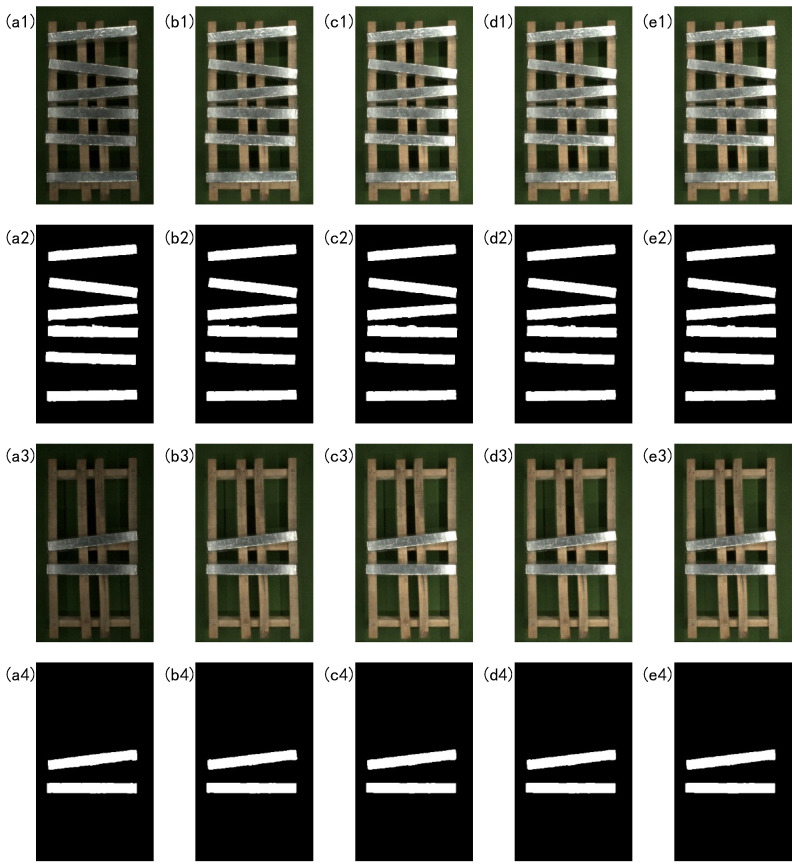
The segmentation results of light source brightness changes. (**a1**–**e1**) and (**a3**–**e3**) are the input images, and (**a2**–**e2**) and (**a4**–**e4**) are the results of image segmentation. From left to right, the brightness of the light source is 50, 100, 150, 200, and 255 (255 is the maximum brightness of the current strip light source).

The intensity of the light source will directly affect the area of the overexposed area on the surface of the magnesium ingot. In order to verify the adaptability of the algorithm in this paper to different overexposed areas and to be able to adapt to the interference of light sources with different intensities, a control experiment is designed for different illumination. The brightness of the light source of the strip light is adjusted through a dimmer controller. And the segmentation results of the magnesium ingot are shown in Figure 21.

As can be seen from the input image in Figure 21, as the brightness of the light source increases, the overexposure area of the magnesium ingot region gradually increases, and the degree of overexposure becomes more severe. From the segmentation results of the magnesium ingot image, the algorithm in this paper is basically not affected by changes in light source brightness, and has good reliability. It can maintain a higher rate of image segmentation under different light source brightness.

(3) Verifying the effectiveness of the algorithm under actual operating conditions.

**Figure 22:**
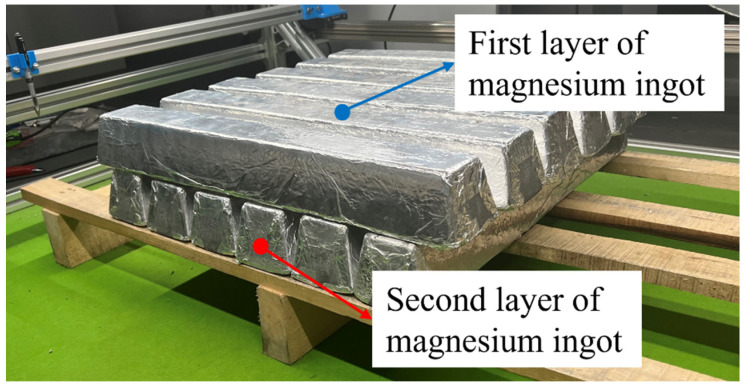
Schematic diagram of double-layer magnesium ingot arrangement.

In an industrial production environment, magnesium ingots are arranged in a vertical cross shape and in multiple layers. Due to the limit of experimental conditions, the number of magnesium ingots is insufficient. Take a double-layer arrangement of magnesium ingots as an example, and the specific arrangement is shown in Figure 22. In addition, during the actual sorting process, magnesium ingots are identified layer by layer, and each layer of magnesium ingots is covered, enabling one-time access to the location information of the entire layer of magnesium ingots. The experiment arranges magnesium ingots according to actual industrial production, and designs a comparison of brightness values of light sources.

**Figure 23:**
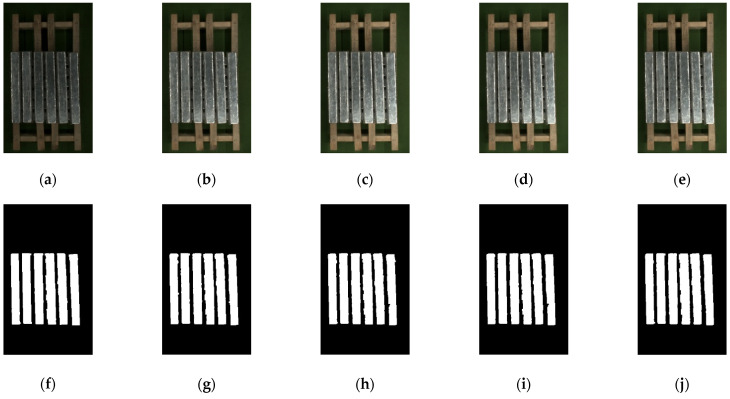
The results of image segmentation under actual operating conditions. (**a**–**e**) are the input double-layer magnesium ingot images, and (**f**–**j**) are the results of image segmentation. From left to right, the brightness values of the light source in the image are 50, 100, 150, 200, and 255.

In Figure 23, it can be seen from the image in the first row that the lower magnesium ingots leaked out in the middle of the magnesium ingot gap. Due to the fixed position of the strip light source, as the number of layers of magnesium ingots increases, the distance from the light source becomes closer and closer, and the overexposure situation on the surface of the magnesium ingot changes. In addition, a comparison of light source brightness value is designed. From the results of image segmentation, the algorithm in this paper can still have the good accuracy of segmentation under the actual working conditions, even if the brightness of the light source changes.

(4) Comparing with other algorithms to verify the reliability of this algorithm.

**Figure 24:**
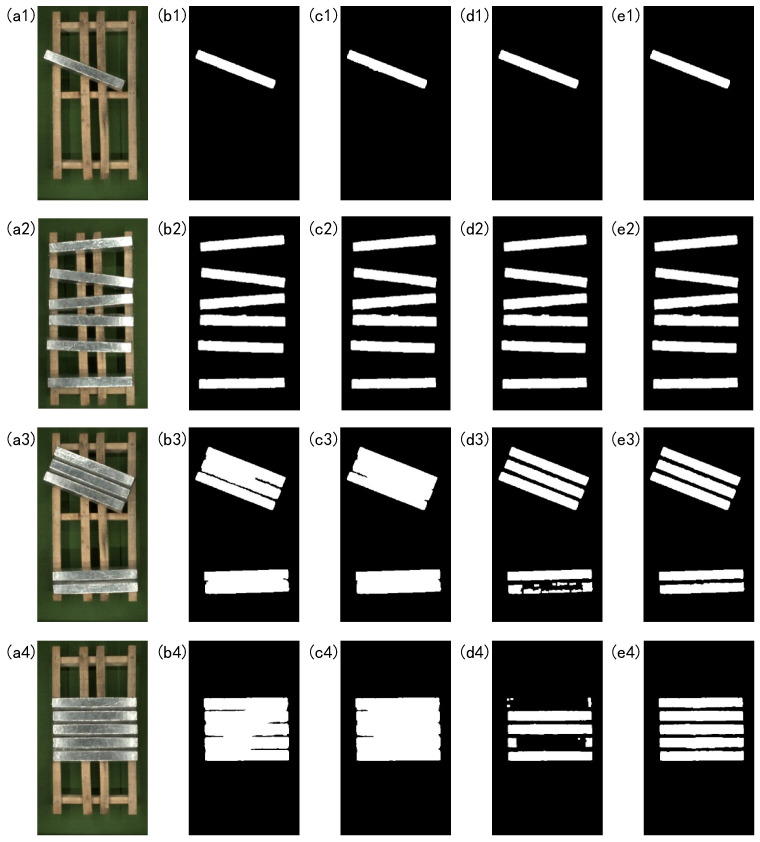
Image segmentation results of different algorithms. (**a1**–**a4**) are the input image, and (**b1**–**b4**), (**c1**–**c4**), (**d1**–**d4**), and (**e1**–**e4**) are the corresponding segmentation results using the Otsu threshold, Triangle threshold, Adaptive threshold, and proposed method.

This experiment selects four magnesium ingot images with different postures and compares the segmentation results of the four algorithms. They are the Otsu threshold [30], Triangle threshold [31], Adaptive threshold, and proposed method. The segmentation results of the experiment are shown in Figure 24.

**Figure 25:**
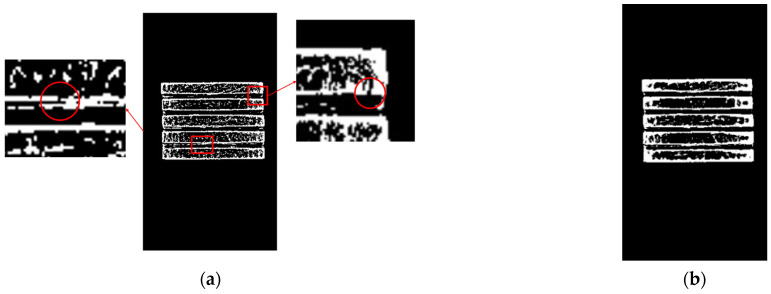
Initial segmentation result of Figure 24(a4). (**a**) The result of the Adaptive threshold. In the red circle, pixels do not have connectivity; (**b**) the result of the proposed method.

From Figure 24 the four segmentation methods can achieve good segmentation results for a single magnesium ingot and when magnesium ingots are separated from each other. When the ingots are closely aligned, in Figure 24(b3,c3,b4,c4), there is an adhesion phenomenon in the segmentation results, which makes it impossible to distinguish between magnesium ingots and magnesium ingot gaps. Therefore, the Otsu threshold and Triangle threshold are not suitable for magnesium ingot segmentation. In Figure 24(d3,d4), magnesium ingots and magnesium ingot gaps are distinguished to some extent, but there are varying degrees of missing phenomena in the segmentation results. The reason is that after processing the image using the Adaptive threshold, the pixels in the initial segmentation result do not have connectivity, resulting in the failure of subsequent morphological processing. The initial segmentation result of Figure 24(a4) is shown in Figure 25. Compared with the Adaptive threshold, the segmentation result of the proposed method is more complete. The proposed method can cope with the changing posture of magnesium ingots with greater accuracy and robustness.

We collected a total of 250 magnesium ingot images, including various light source brightness, various magnesium ingot postures, and magnesium ingot arrangement methods. When each magnesium ingot in the image is recognized independently, the image is considered as a fully recognized image. The calculation for the segmentation accuracy in this paper is the percentage of fully recognized images out of the total number of captured images. Comparative experiments were conducted between the three threshold segmentation algorithms and ours, and their segmentation accuracies are shown in Table 1. Because the experimental image contains multiple magnesium ingot arrangements, the Otsu threshold and Triangle threshold cannot accurately distinguish between magnesium ingots and magnesium ingot gaps, and their segmentation accuracies are unsatisfactory. Compared with the Adaptive threshold, the segmentation accuracy of the proposed method has significantly improved.

From the collected images, a total of 225 images were selected. They were divided into two groups of experiments according to different the postures and light source brightness of magnesium ingot images, comparing the recognition rates of the four methods.

**Figure 26:**
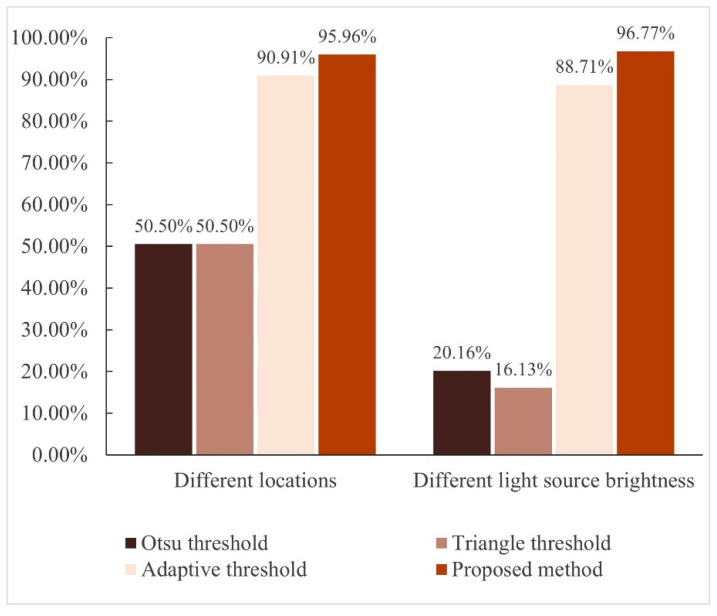
Comparison histogram of the four algorithms.

In Figure 26, the Otsu threshold and Triangle threshold have unideal recognition rates in experiments and cannot meet normal industrial needs. Due to changes in the brightness of the light source, the overexposure on the surface of the magnesium ingot will change, increasing the difficulty of segmentation, and significantly reducing the recognition rate. In experiments with different locations of magnesium ingots as variables, the recognition rates of the Adaptive threshold and the proposed method in this paper are both relatively high, reaching 90.91% and 95.96%, respectively; however, in experiments with different light source brightness as variables, the recognition rate of the Adaptive threshold is only 88.71%, while the recognition rate of the proposed method can reach 96.77%, which proves that the proposed method has strong anti-interference ability in the face of changes in ambient luminance, and the overall recognition rate is high.

In addition, during the magnesium alloy smelting process, the basic production requirement is to deliver two magnesium ingots per minute. The real-time requirement for the identification process is not high, and our current identification efficiency can meet the production requirements.

## 4. Conclusions

This algorithm can be applied to the recognition of magnesium ingots with high reflectivity on the surface, solving the segmentation difficulties caused by overexposure of magnesium ingots due to changes in lighting. This paper proposes an algorithm for detecting and correcting overexposed regions for high reflection of magnesium ingots, and designs an adaptive threshold magnesium ingot stack segmentation algorithm based on image overexposed region correction. This algorithm identifies overexposed areas in an image through the mapping relationship between image brightness and chrominance information, and corrects them based on their neighborhood color information. The corrected image effectively improves segmentation accuracy, and this algorithm can effectively improve the antienvironmental interference ability of image segmentation processing.

In the experiment presented in this paper, we achieved satisfactory segmentation results for the magnesium ingots with our current shape and size under the current lighting environment. However, in order to obtain even better experimental results, we plan to further explore the segmentation of magnesium ingots with different shapes and sizes under more complex lighting conditions.

## Figures and Tables

**Table 1:** The results of algorithm recognition.

Method	Segmentation Accuracy
Otsu threshold	30.12%
Triangle threshold	28.11%
Adaptive threshold	86.34%
Proposed method	94.38%

## Data Availability

Not applicable.
